# Beta-blockers and the treatment of hypertension: it is time to move on

**Published:** 2007

**Authors:** Charles Shey Wiysonge, Jimmy Volmink, Lionel H Opie

**Affiliations:** South African Cochrane Centre, Medical Research Council, Cape Town; Faculty of Health Sciences, University of Stellenbosch; South African Cochrane Centre, Medical Research Council, Cape Town; Hatter Cardiovascular Research Institute, Department of Medicine, University of Cape Town

Existing solid scientific evidence with hard outcome data should be the basis for treatment guidelines, and where such evidence is lacking, we must invest in research.^1^ A case in point is the initiation of antihypertensive treatment with a beta-blocker.^2^-^4^

Beta-blockers are pharmacological agents that block the action of endogenous catecholamines on beta-adrenergic receptors, part of the sympathetic nervous system which mediates the ‘fight or flight’ response. The main adrenergic receptors present in human cardiovascular tissues are the β_1_-, β_2_-, and α_1_-receptors.^5^ β_1_-adrenergic receptors are located mainly in the heart and kidneys and β_2_-receptors are found mainly in the lungs and gastrointestinal tract. The α_1_-receptors mediate endothelial function and vasoconstriction in peripheral blood vessels and regulate blood flow to the kidney.

Beta-blockers differ with regard to β_1_/β_2_-adrenergic receptor selectivity and vasodilatory activity, and these differences have led to their subdivision into first-, second- and third-generation agents. First-generation beta-blockers, such as propranolol and pindolol, are termed non-selective since they exert equal blockade of β_1_- and β_2_-receptors. The second-generation beta-blockers (such as atenolol and metoprolol) are described as selective because they exhibit higher affinity for β_1_- than β_2_-adrenergic receptors. Finally, third-generation beta-blockers (eg, carvedilol and nebivolol) differ from first- and second-generation betablockers in their vasodilatory properties.^5^

Beta-blockers have been routine treatment for patients with hypertension for several decades, apparently because activation of the sympathetic nervous system is important in the aetiology and maintenance of hypertension.^6^ We recently re-assessed the effectiveness and safety of these pharmacological agents when used as first-line treatment for hypertension.^3^,^4^ Evidence from randomised, controlled trials published by 1992 show that hypertensive patients who were treated with a first- to second-generation beta-blocker for a median duration of about five years had their relative risk (RR) of stroke and all cardiovascular events reduced by 20% [95% confidence interval (CI) 4–34%] and 12% (95% CI 3–21%), respectively, compared to those on placebo or no treatment. These effects of beta-blockers were similar to those of thiazide diuretics, but patients were more likely to withdraw from a beta-blocker due to the side effects than a diuretic (RR 86%, 95% CI 39–150%).

However, between 2002 and 2005, scientific evidence rapidly accumulated to show that the cardiovascular protection and safety profile of beta-blockers was inferior to that of newer antihypertensive agents such as calcium channel blockers and inhibitors of the renin-angiotensin system. The incidence of stroke was significantly higher for patients whose antihypertensive treatment was commenced with a beta-blocker than for those who received a renin-angiotensin system inhibitor [relative risk increase (RRI) 30%, 95% CI 11–53%] or a calcium channel blocker (RRI 24%, 95% CI 11–40%). In addition, the risk of death from any cause (RRI 7%, 95% CI 0–14%) and any cardiovascular event (RRI 18%, 95% CI 8–29%) was higher for patients on beta-blockers than those on calcium channel blockers. 3 It has also been shown that beta-blockers significantly increase the risk of new-onset diabetes compared to placebo (RRI 25%, 95% CI 5–50%), renin-angiotensin system inhibitors and calcium channel blockers.^7^

When medication costs and the costs associated with treatment of hypertension-related and antihypertensive-induced complications are considered, beta-blockers are less cost-effective than thiazide diuretics, renin-angiotensin system inhibitors and calcium channel blockers.^8^

It is important to note that the current evidence derives mainly from trials of first- and second-generation beta-blockers (mainly atenolol), as there are no outcome data yet on third-generation beta-blockers.3 The sub-optimal cardiovascular protection with conventional (ie, first- and second-generation) beta-blockers may be due to the development of new-onset diabetes and the inability to decrease central aortic pressure as much as brachial pressure.^9^ In theory, third-generation beta-blockers should reduce central blood pressure more than conventional beta-blockers because vasodilatation by the former may alter the pattern of the pressure wave reflecting back from the periphery.^9^,^10^ In addition, the newer beta-blockers may have a better metabolic profile.^10^

Clinicians should use the currently available scientific evidence^3^,^7^,^8^ to guide the management of their patients with hypertension but this does not yet seem to be the case. Betablockers are still widely used worldwide. For example, 12 to 29% of patients on antihypertensive drugs in various European countries are on beta-blockers, a substantial proportion on atenolol.^11^ We think it is now time to move on. There is a need for long-term, outcome-randomised, controlled trials to compare the effects of third-generation beta-blockers^10^ with those of renin-angiotensin system inhibitors and calcium channel blockers. In the meantime, guideline developers should no longer recommend beta-blockers for initiating antihypertensive treatment.

Similarly, conventional beta-blockers should no longer be used as comparator drugs in randomised, controlled hypertension trials. We do, however, acknowledge that some patients with hypertension may require beta-blockers for symptomatic angina, chronic stable heart failure and post-myocardial infarction protection, or as part of multiple therapy for resistant hypertension.^8^,^12^,^13^

The United Kingdom National Institute for Health and Clinical Excellence and the British Hypertension Society have taken the bull by the horns and downgraded beta-blockers from first- to fourth-line antihypertensive drugs, ie, add-on drugs in patients requiring multiple therapy.^8^ The South African Hypertension Society has made a similar recommendation.^14^ While other hypertension guidelines have not (yet) been updated in the light of the current evidence,^15^ the European Society of Hypertension and the European Society of Cardiology still overlook the current evidence and recommend the use of any antihypertensive agent (including beta-blockers) for initiation and maintenance of antihypertensive treatment, alone or in combination. 16 However, the American Heart Association has just recommended that for patients at high risk of coronary artery disease, such as those with diabetes, chronic renal disease, or a 10-year Framingham risk score of 10% or higher, first antihypertensive choices should exclude beta-blockers.^17^

In summary, beta-blockers are effective in preventing cardiovascular disease but are no longer suitable for routine initial treatment of hypertension because their cardiovascular protection and metabolic effects are worse than those of other antihypertensive drugs. However, it is time to move on, and randomised, controlled, hypertension outcome trials are needed to prove the non-inferiority of the newer vasodilating beta-blockers (such as nebivolol and carvedilol) in comparison with renin-angiotensin system inhibitors and calcium channel blockers.
